# Directional quantum dot emission by soft-stamping on silicon Mie resonators†

**DOI:** 10.1039/d1na00630d

**Published:** 2022-01-07

**Authors:** Tom Veeken, Benjamin Daiber, Harshal Agrawal, Mark Aarts, Esther Alarcón-Lladó, Erik C. Garnett, Bruno Ehrler, Jorik van de Groep, Albert Polman

**Affiliations:** Center for Nanophotonics, NWO-Institute, AMOLF Science Park 104 1098 XG Amsterdam The Netherlands t.veeken@amolf.nl; Institute of Physics, University of Amsterdam Science Park 904 1098 XH Amsterdam The Netherlands

## Abstract

We present a soft-stamping method to selectively print a homogenous layer of CdSeTe/ZnS core–shell quantum dots (QDs) on top of an array of Si nanocylinders with Mie-type resonant modes. Using this new method, we gain accurate control of the quantum dot's angular emission through engineered coupling of the QDs to these resonant modes. Using numerical simulations we show that the emission into or away from the Si substrate can be precisely controlled by the QD position on the nanocylinder. QDs centered on a 400 nm diameter nanocylinder surface show 98% emission directionality into the Si substrate. Alternatively, for homogenous ensembles placed over the nanocylinder top-surface, the upward emission is enhanced 10-fold for 150 nm diameter cylinders. Experimental PL intensity measurements corroborate the simulated trends with cylinder diameter. PL lifetime measurements reflect well the variations of the local density of states at the QD position due to coupling to the resonant cylinders. These results demonstrate that the soft imprint technique provides a unique manner to directly integrate optical emitters with a wide range of nanophotonic geometries, with potential applications in LEDs, luminescent solar concentrators, and up- and down-conversion schemes for improved photovoltaics.

## Abstract 2

We present a soft-stamping method to selectively print a homogenous layer of CdSeTe/ZnS core–shell quantum dots (QDs) on top of Si nanocylinders with Mie-type resonant modes. Depending on the cylinder shape, we direct the QD emission up or down.

## Graphical abstract

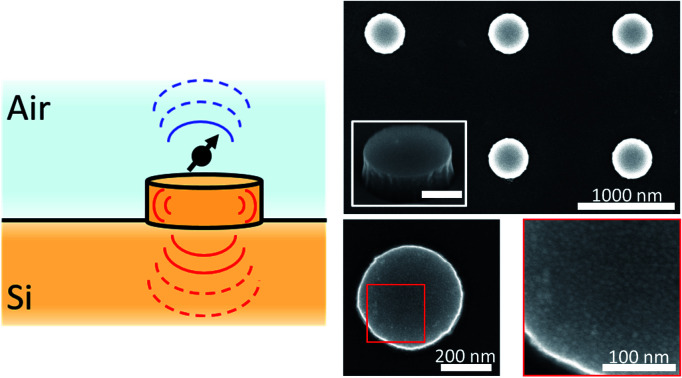

## Introduction

Accurate control over the angular distribution of light emission is of great importance in many technological applications. In light-emitting diodes (LEDs) for example, controlling the angular distribution of light emission inside the semiconductor into the escape cone optimizes the output power.^1,2^ In solid-state lighting systems, tailored visual appearances can be achieved by controlling the angular distribution of light emission.^3,4^ And in photovoltaic systems, control over the directionality of emission can improve the efficiency of luminescent solar concentrators^5,6^ and enhance the efficiency of up- and down-conversion schemes.^7–10^

Resonant nanostructures can help tailor the emission of dipole-like point emitters by controlling the coupling between the resonant modes and the emitter. The spectrum, polarization, and angular distribution of the emission are then determined by the coherent superposition of the scattered fields of the electric and magnetic multipoles and their coupling to the emitter's dipole moment. Initial work in this area focused on coupling noble-metal nanoparticles to optical emitters, where the spectrum and polarization were controlled by coupling emitters to selected plasmonic modes^11,12^ and directional emission was achieved with nanoparticle antenna's.^13–16^ More recently, all-dielectric resonant nanostructures have received great interest because of their strongly reduced optical losses and the larger variation of multipoles that can be excited.^17–20^ This offers more degrees of freedom to design the resonant interaction with the emitter. Moreover, optical emitters can be placed directly inside the resonant nanostructures.^4,21^*Vice versa*, nanostructures can be directly placed on top of emitters, as recently shown for resonant silicon (Si) nanowires on monolayers of MoS_2_,^22^ to create directional forward and backward emission depending on the complex interplay of the nanowire resonances. In these pioneering first experiments, there was only limited control on the exact placement of emitter with respect to the nanoresonator.^23–25^ However, to leverage all benefits of the dielectric resonator–emitter coupling, precise control over the placement and coupling between the emitter and resonant nanostructure is of great importance. Selective coating of nanostructures with uniform monolayers of emitters in particular would be highly desirable for applications in *e.g.* LEDs and luminescent concentrators.

Here, we introduce a new method to achieve selective control over the placement of optical emitters on resonant nanostructures using a soft stamping technique.^26^ As a demonstration, we fabricate arrays of Si nanocylinders that exhibit strong optical Mie resonances using standard electron-beam lithography (EBL) and reactive-ion etching (RIE) techniques. Then, using a PDMS rubber stamp we selectively place luminescent quantum dots (QDs) on top of nanophotonic structures only in a controlled manner. By directly spin-coating the QDs on the PDMS stamp, we realize a single-step stamping process that is simplified compared to existing pick-and-place stamping techniques.^27–30^ Using photoluminescence (PL) mapping spectroscopy and lifetime measurements in combination with numerical modeling, we show evidence of strong directional emission of the QDs coupled to the nanoresonators. Our results contribute towards novel routes for improved efficiencies of quantum dot applications in LEDs, wide spectrum emission, and enable up and down-conversion schemes in photovoltaics. The resonant directional light emission demonstrated in this work is generic and can be applied to a wide range of emitters, including semiconductor quantum wells, fluorescent molecules, and perovskite films.

## Nanophotonic design

**Fig. 1:**
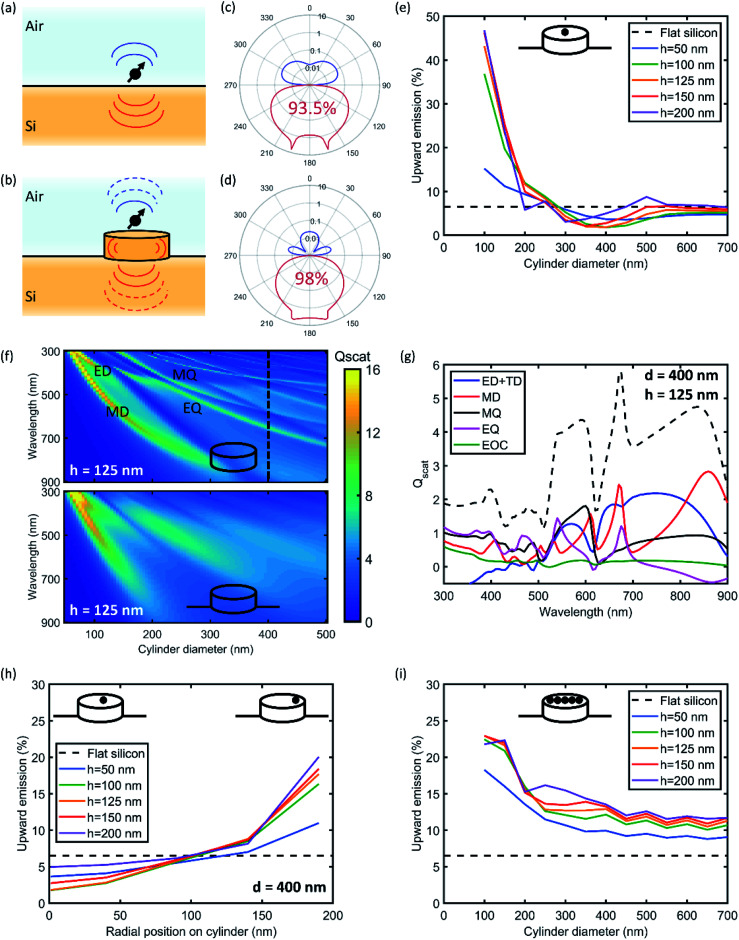
Directional dipole emission by nanophotonic engineering. (a and b) Schematics of a dipole above a Si surface in air and on top of a Si nanocylinder. (c and d) Calculated azimuthal angular dipole emission patterns corresponding to the cases depicted in (a and b) on a logarithmic radial axis. 98% downward emission is achieved for a dipole on top of a cylinder with optimized dimensions. (e) Calculated fraction of upward emission at *λ* = 760 nm as a function of cylinder diameter (*x*-axis) for different heights for a single dipole at the center of the cylinder. (f) Calculated *Q*_scat_ (color) as a function of wavelength and cylinder diameter for a cylinder in air (top) and a cylinder on a Si substrate (bottom). (g) *Q*_scat_ (dashed black) corresponding to the dashed line in (f) and its multipole decomposition (legend). (h) Calculated fraction of upward emission, similar to (e), but now as a function of dipole position on top of the 400 nm diameter cylinder. (i) Calculated fraction of upward emission, similar to (e), but now averaged over a homogeneous distribution of dipole positions on top of the cylinder. For all figures throughout the paper, the distance between the dipole and the Si surface is kept constant at 5 nm, and the dipole emission intensity is averaged over all polarizations. Dashed line indicates the reference case for a dipole on a flat Si surface.

The angular emission intensity distribution in the far field of an optical emitter depends on the local density of optical states (LDOS), given the position and orientation of the emitter and the (near-field) coupling to the available optical modes. The LDOS varies strongly with position near an interface, and the modal density in the far field is linearly proportional to the refractive index.^31^ As a result, an optical emitter in a low-index medium close to a high-index substrate (Fig. 1a) shows strongly anisotropic emission towards the higher index medium.^32^ For distances on the order of the wavelength, back-action between the dipole and its emitted light reflected at the interface leads to strong modulation of the anisotropic emission with distance from the interface, as first shown by Drexhage.^33^ In Fig. 1c, the azimuthal angular emission profile is shown for an electric dipole placed 5 nm above a Si substrate, obtained from an analytical Green's function model.^31^ To emulate the profile of an ensemble of dipoles with a random orientation distribution, as is often the case experimentally, the emission profile in Fig. 1c is the average of the three orthogonal transition dipole moments, *x*, *y*, and *z*. The results in Fig. 1c show that the strong index contrast at the Si/air interface results in 93.5% of the emission being directed into the Si substrate. For photovoltaic applications, this 6.5% loss is significant and therefore requires enhancing the downward emission even further. On the contrary, for light-emitting applications, the anisotropy caused by a high index substrate calls for enhancing the upward emission. Both these effects are addressed in this paper.

To control the emission anisotropy, high-index resonant nanostructures are placed in the near field of the emitter, as depicted in Fig. 1b. Silicon structures with dimensions in the few-100 nm range support an electric dipole (ED), magnetic dipole (MD), and higher-order multipoles.^17,34^ The coupling of the emitter to these multipolar modes alters the angular emission pattern through interference of the scattered light in the far-field. The shape and size of the resonator determines the strength of the multipolar modes at the emission wavelength, and as such, acts as a control for the direction of emission. We first use Finite-Difference Time-Domain (FDTD) simulations to calculate the coupling between the emitter and the nanostructure, using known optical constants for Si and a dipole emitting at *λ* = 760 nm placed 5 nm above the center of a Si cylinder. In Fig. 1e, the fraction of radiation emitted into the top hemisphere is plotted as a function of Si cylinder diameter for different heights (see Fig. S1† for corresponding emission profiles). The upward emission is defined as the total radiation emitted into the upper hemisphere, by a single dipole on top of a single cylinder. The dashed line in Fig. 1e indicates the reference value of 6.5% for the case of a substrate without resonator. We find that for small cylinder diameters between 50 and 250 nm, the upward radiation away from the substrate is enhanced, whereas the downward radiation is enhanced in the range of 250–500 nm. Beyond a diameter of 500 nm, the upward emission approaches the reference; here, the Si cylinder has many resonant modes at the emitter wavelength, but none with significant strength, such that the coupling is similar to that for a planar film. The maximum fraction of downward radiation is achieved for a cylinder diameter of 400 nm and height of 125 nm, at a value of 98%. The corresponding azimuthal angular emission profile is shown in Fig. 1d.

To assess which Mie-like multipolar modes are excited in the Si cylinder, we calculate the normalized scattering cross-section *Q*_scat_ using FDTD simulations, defined as the scattering cross-section normalized to geometrical cross-section for a normal-incident plane wave.^17^Fig. 1f shows *Q*_scat_ as a function of wavelength and cylinder diameter. Many Mie-like resonances are visible for the cylinder without substrate (top), some of which can be attributed to single multipole resonances such as the ED, MD, toroidal dipole (TD), electric quadrupole (EQ), and magnetic quadrupole (MQ). When the substrate is introduced under the cylinder (Fig. 1f, bottom), the lineshape of the multipolar resonances broadens significantly due to strong radiative leakage from the resonant mode into the substrate. However, the *Q*_scat_ remains well above unity, indicating the strong resonant character of the cylinder.

The broad range of Mie-like modes in a Si cylinder is shown in Fig. 1g, for the cylinder with maximum emission downwards but without the substrate. To obtain insight in which modes contribute to the strongly directional radiation pattern, we use a multipole decomposition to extract the relative contribution of a set of multipolar resonances: ED and toroidal dipole (TD), MD, EQ, MQ, and the electric octupole (EOC).^34^ Clearly, all five components attribute to the *Q*_scat_ over a broad range of wavelengths, and notably quite evenly at the target wavelength of 760 nm. Therefore, we attribute the enhanced downwards emission to a combination of multipolar modes in the cylinder and direct emission of the dipole into the far-field. Note that the multipolar resonances we find here are those that can be excited by the normal-incident plane wave. A dipole-like point emitter placed in the near-field of the nanocylinder can couple to the resonant modes with different relative amplitudes, and also excite modes with symmetric modal field profiles that cannot be excited a plane wave. Despite this, the multipole analysis provides valuable insight in the complex combination of multipolar resonances that collectively give rise to strong broadband light scattering.

So far, we analyzed the emission of a dipole placed at the center of the cylinder's top surface. Now, we investigate the angular emission for an ensemble of dipoles homogeneously distributed over the nanocylinder surface. We use FDTD to calculate the angular emission distribution for different radial positions on the nanocylinder surface, as shown in Fig. 1h for the cylinder diameter of 400 nm (which showed the highest downward emission in Fig. 1e). Subsequently, we average these simulation results, weighted by their radial area, to determine the upward emission fraction for the ensemble, which is shown in Fig. 1i. The result shows that the average upward directionality is always higher than that for the flat reference, *i.e.*, the upward emission can be tuned. Similar to the calculation for the single emitter at the center, the curves converge to the reference for large diameter, but there is no cylinder geometry where the emission reaches below the reference. This means that emitters placed in the outer perimeter of the nanocylinder surface couple well to multipolar resonances that promote upward emission (see Fig. S2†). Fig. 1h and i indicate that the placement of quantum dots on the nanocylinders strongly controls the directional emission.

## Fabrication: soft-stamping of QDs onto nanostructures

**Fig. 2:**
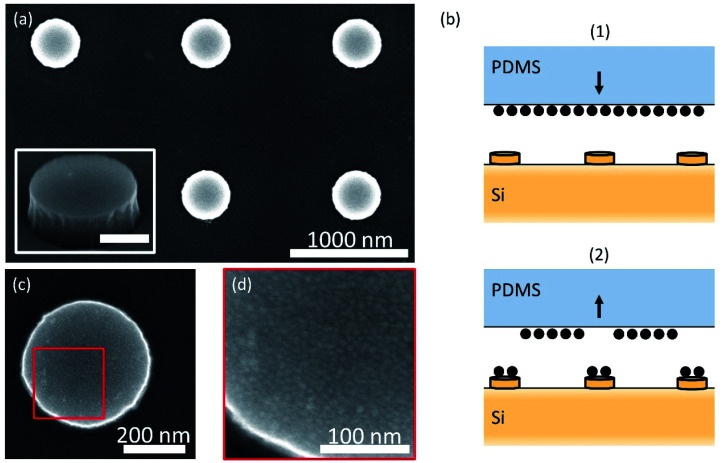
Nanocylinder fabrication and selective QD stamping. (a) SEM micrograph of Si cylinders on a Si substrate fabricated with EBL and RIE. Inset: SEM micrograph under an angle, showing straight sidewalls (scale bar is 200 nm). (b) Schematic representation of the stamping procedure; (1) QDs are spin-coated on a silanized PDMS stamp and pressed down on the nanostructured Si substrate; (2) after pressing for 10 minutes at 3 N force and 40 °C, the stamp is retracted, leaving behind the QD layer that came into contact with Si. (c and d) High-resolution SEM micrograph of Si cylinders after QD stamping showing a complete and conformal layer of QDs on the surface.

To experimentally demonstrate the nanophotonic control over QD directional emission, we fabricated the structures designed above. Silicon nanocylinders were patterned into the top surface of a Si substrate by EBL. A negative tone resist, hydrogen silsesquioxane (HSQ), was spin-coated on top of a polished Si(100) substrate (500 μm thick). Square arrays of disks were exposed in the HSQ with the electron beam. After development, only the exposed HSQ remained, which was subsequently used as an etch mask during the RIE step. A scanning electron microscopy (SEM) image of the resulting Si cylinder arrays is shown in Fig. 2a for a target diameter of 425 nm, height of 145 nm, and pitch of 1275 nm. This design differs slightly from the optimal design in Fig. 1e because fabrication was based on designs at a different target emission wavelength.

Subsequently, QDs were selectively placed on top of the Si cylinders using our novel one-step soft stamping procedure as depicted in Fig. 2b. We used CdSeTe/ZnS core–shell QDs (QDot 800, Thermo Fisher) dispersed in octane. First, a layer of QDs was directly spin-coated on top of a silanized PDMS stamp. The Si nanocylinder sample surface was activated with a UV-ozone treatment. Then, the PDMS stamp was mounted above the Si sample in a soft printing machine, face down. The stamp was brought into contact with the sample and pressed down with a force of 3 N. Once in contact, the sample was heated to 40 °C for 10 min, after which the stamp was withdrawn. The stamping was implemented with motorized controls. The flexible nature of PDMS facilitated a conformal contact with the substrate, which printed a uniform film of QDs on the sample. Fig. 2c shows a representative SEM image of the top surface of a cylinder after stamping, showing the homogeneous coverage. In the zoomed-in image (Fig. 2d), the individual QDs can be recognized with a diameter of ∼10 nm on the cylinder surface. The QD print on the cylinder and the clean Si surface besides the cylinder in Fig. 2c confirm that the stamping method printed completely and selectively on top of the tallest nanostructured surface.

## Photoluminescence measurements

**Fig. 3:**
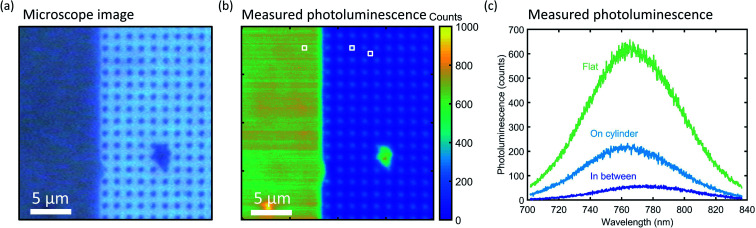
Photoluminescence mapping after thick QD print. (a) Microscope image of the Si substrate (blue) with a thick QD layer on top of flat Si and the Si cylinder array (gray). (b) PL map of the area depicted in (a). Color scale depicts the intensity at 760 nm wavelength. (c) PL spectra of three typical positions on the sample, indicated by the squares in (b): on the flat Si (green), in the middle of a cylinder (light blue), and in between four cylinders (dark blue).

To characterize the directionality of the QD emission, we use a WITec confocal microscope in reflection mode. Fig. 3a shows an optical image of the edge of a cylinder array using a 100× magnification and a broadband LED illumination source. The dark area on the left is the flat Si surface covered by a film of QDs. On the right, the dark dots correspond to the cylinders with a QD layer on top, while the bare Si substrate in between shows a brighter reflection.

Fig. 3b shows a photoluminescence (PL) intensity map at *λ* = 760 nm of the same region imaged in Fig. 3a. A PL spectrum was measured under excitation of a *λ* = 532 nm laser at each position on the map. The measured PL intensity in the upwards direction is defined by the collection of the objective, *i.e.* up to angles of ∼64 degrees (NA = 0.9) from the confocal collection spot of 1 μm. Clear QD emission is observed on top of the cylinders, and strong emission is also observed from QDs printed on the Si wafer next to the nanopatterned region. The latter directly results from the use of a flexible stamp that conformally coats the surface. In contrast, in between the cylinders, the signal is low. In Fig. 3c, the PL spectra for three characteristic positions are plotted: on the flat Si surface, on top of a cylinder, and in between four cylinders. The characteristic wide-band QD emission spectrum is observed in all three cases. We attribute the emission observed in the map between the cylinders to the fact that the tail of the laser (diffraction-limited spot size ∼300 nm) excites QDs on top of the cylinders when the spot is centered in between cylinders and indirect excitation by light scattered from small roughness on the etched Si surface.

**Fig. 4:**
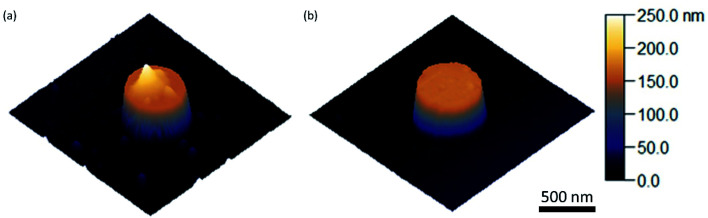
AFM height mapping; 1.5 × 1.5 μm^2^ maps. (a) AFM image of a single cylinder with a thick layer of QDs stamped on top. The print is non-uniform and incomplete. (b) AFM image of a single cylinder with a monolayer of QDs stamped on top. The print is uniform and complete.

The PL data in Fig. 3 have been obtained for a rather thick QD layer printed on the sample. This results in a strong signal in the PL map in Fig. 3b and strong contrast between the cylinders and the surface, enabling direct imaging of QDs on top of the cylinders. Atomic force microscopy (AFM) measurements of the printed layer are shown in Fig. 4a. The printed layer is not completely conformal: the top surface of the Si cylinder is clearly visible (dark orange), with the inhomogeneous QD coverage in brighter colors, up to a height of 100 nm. To perform optical experiments on a thinner and more homogeneous QD layer and to enable a comparison with our simulation results, we repeated the printing process with a QD monolayer spin-coated on the PDMS stamp. An AFM map of the resulting QD coverage is shown in Fig. 4b, showing a homogeneous thin film of QDs on the cylinder surface. This imprint of a thin film of QDs corresponds to the SEM images in Fig. 2b and c.

**Fig. 5:**
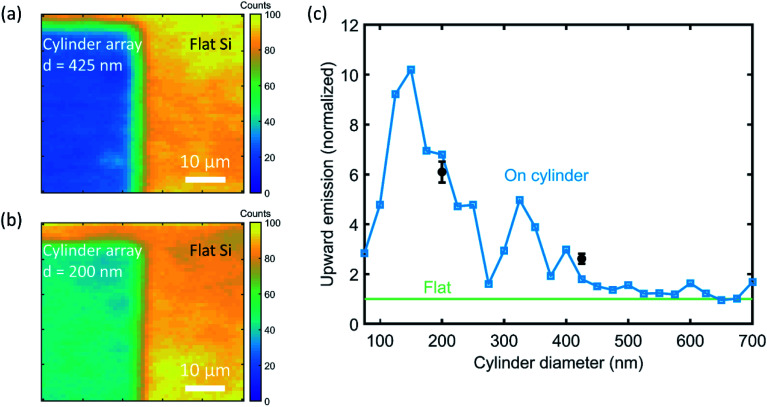
Photoluminescence mapping after monolayer QD print. (a and b) PL maps of the boundary area between a cylinder array and flat Si, for diameters of 425 and 200 nm, respectively. Color scale depicts the intensity at *λ* = 760 nm. (c) Calculated upward emission intensity (blue) as a function of cylinder diameter at the experimental cylinder height (145 nm), averaged over the cylinder surface. Calculations are corrected for resonant excitation enhancement of the QDs at the cylinder surface at the laser wavelength and normalized to the flat Si reference case (green) to emulate experimental conditions. Experimental data points (black) corresponding to measurements in (a and b) are in good agreement with the predictions.

Fig. 5a shows the PL map for the same cylinder array as in Fig. 3, with 425 nm diameter and 1275 nm pitch, but now with a QD monolayer on top. The emission from the individual cylinders is not distinguishable here because of the smaller 20× magnification. To compare the QD emission from the cylinder array with the flat Si beside it, we correct the emission counts for the ratio of the unit cell area to the cylinder top area, assuming that the cylinders are covered with QDs and that there are no QDs between the cylinders. We find that the upward emission intensity from the QDs on the cylinders, as collected by the microscope, is enhanced by a factor 2.6 ± 0.2 relative to the flat Si reference.

To compare the measured results with simulations, we have to account for resonant enhancement of the quantum dot by the cylinders. To do this, we derive the excitation enhancement by using FDTD to obtain the electric field enhancement above the cylinder at the laser wavelength (see Methods and Fig. S3†). The electric field intensity above the cylinder is then used to weigh the contribution to the upward emission intensity of each position on the cylinder's top surface. From this analysis, we find an upward emission enhancement by a factor 1.8, close to the experimental value found above from Fig. 5a. This confirms that the FDTD simulations accurately predict the coupling between the emitter and the nanostructure in the near field and the resulting far field emission.

We now use FDTD to calculate the upward QD emission intensity as a function of cylinder diameter, normalized to the flat Si reference simulation (Fig. 5c). A strong dependence on cylinder diameter is observed, with a ten-fold enhancement for 150 nm diameter cylinders. To test this experimentally, we measured a PL map for a cylinder diameter of 200 nm and pitch of 600 nm (Fig. 5b). The same analysis as for Fig. 5a yields an enhancement factor for upward emission of 6.1 ± 0.4, consistent with the upward trend for smaller diameter shown in the simulations of Fig. 5c, but below the calculated value of 6.8. We explain the discrepancies by small differences in geometry between experiment and calculation. Overall, the experimentally observed enhanced upward emission is well explained by the combination of resonant directional emission and enhanced excitation obtained from simulations. Fig. 5c doesn't compare to the results of Fig. 1 due to the correction for the excitation enhancement; separate curves for the simulated upward emission and excitation enhancement can be found in Fig. S5.†

## Photoluminescence lifetimes

**Fig. 6:**
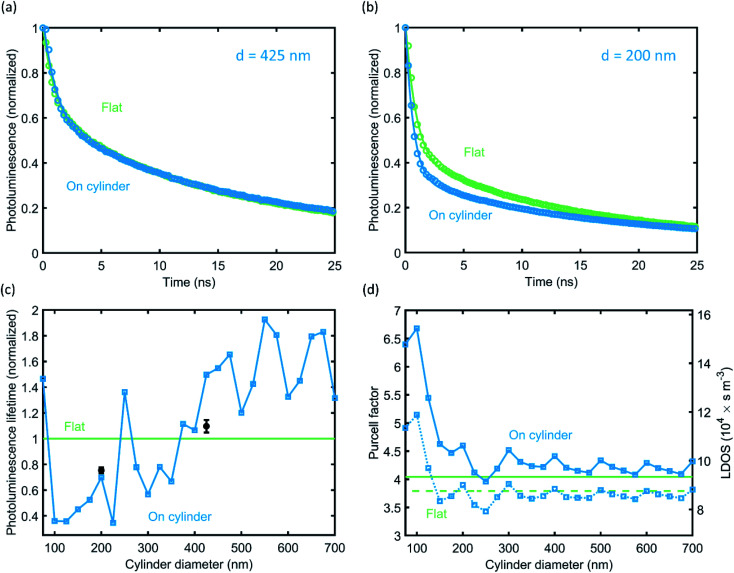
PL lifetime measurements after monolayer QD print. (a and b) Measured (circles) and fitted (lines) PL decay traces on a cylinder array (blue) and on flat Si (green), for cylinder diameters of 425 and 200 nm, respectively. (c) Calculated PL radiative lifetime (blue) as a function of cylinder diameter at the experimental cylinder height (145 nm), averaged over the cylinder surface. Average calculations account for the resonant excitation enhancement of the QDs due to the cylinders at the laser wavelength and are normalized to the flat Si reference (green) to emulate experimental conditions. Experimental data points (fast decay components, black) from the measurements in (a and b) are in good agreement with the calculations. (d) Calculated Purcell factor (left *y*-axis) and corresponding LDOS values (right *y*-axis) as a function of cylinder diameter, averaged over the cylinder surface (blue). Flat Si reference case (green) is indicated, as well as the downward emission fractions (dashed lines).

To corroborate the role of resonant coupling to optical modes in the nanocylinders in the emission directionality, we study the PL emission lifetime. PL lifetime measurements are conducted using a time-correlated single-photon counting (TCSPC) setup with 485 nm excitation wavelength (see Methods). Fig. 6a and b shows the decay traces for measurements on the 425 nm and 200 nm cylinder arrays, respectively, and on the flat Si substrate directly besides it. Clearly, QD emission on the 200 nm cylinder decays faster than that on the flat Si beside it, while the decay for the 425 nm cylinder is nearly identical to that for the flat Si. The decay traces were fitted with a sum of two exponentials, shown as the line through measured the data points in Fig. 6a and b. The lifetime values obtained from this fitting procedure are detailed in Table 1. We assign the faster decay for the 200 nm diameter cylinders to the enhanced LDOS due to the strongly modified nanophotonic environment provided by the cylinders.

**Table 1:** PL lifetimes corresponding to the fitted curves in Fig. 6a and b

	Flat silicon		On cylinder	
	Fast lifetime	Slow lifetime	Fast lifetime	Slow lifetime
*d* = 425 nm	1.11 ns	20.1 ns	1.22 ns	21.7 ns
*d* = 200 nm	0.91 ns	18.8 ns	0.69 ns	21.3 ns

To analyze this in more detail, we use the FDTD simulations of the angular emission profiles to calculate the PL emission rate (see Methods). Fig. 6c shows the simulated PL radiative lifetime for a dipole on a Si cylinder, normalized to the lifetime of a dipole on a flat Si substrate and averaged over all possible dipole positions. Here we also correct for the excitation enhancement by the laser on the cylinder (see Fig. S5†), to ensure that the simulations emulate the experimental conditions. A strongly varying dependence of cylinder diameter is observed, and the measured decay rates (black) correspond well with the simulations: for the 200 nm diameter cylinder, the lifetime is significantly shorter than for the flat Si reference case, while the lifetime of the 425 nm diameter case is just above that for the reference. The variations in the simulated lifetime are almost entirely due to the simulated excitation enhancement (see Fig. S6†), which further corroborates the necessity of the correction.

Finally, we investigate the Purcell factor and LDOS as a function of cylinder diameter. The PL lifetime curve in Fig. 6c results from the convolution of the calculated LDOS enhancement and laser excitation enhancement, which corresponds to the measurement conditions and compares well with the results. In Fig. 6d, we leave out the correction for the laser excitation enhancement and plot only the calculated emission enhancement as a function of the cylinder diameter (but still averaged over polarization and cylinder surface). The left and right *y*-axes values are the Purcell factor and absolute LDOS, respectively, which directly follow from the FDTD calculations and the free-space LDOS at the emission wavelength (see Methods). We find an increase of the Purcell factor of about 10% for almost all diameters compared to the flat Si reference. The dashed lines in Fig. 6d depict the downward emission fraction for the cylinder and reference case. Comparing the total LDOS to the downward LDOS and both reference values, we can conclude that the enhanced upward emission (Fig. 1i) is due to an increase in the upward LDOS – the downward LDOS is almost equal to the reference. For diameters of 150 nm and smaller, the LDOS increases significantly while simultaneously the upward emission fraction increases, detailing that the cylinder modes increase the upward LDOS more strongly. The LDOS as a function of dipole position on the cylinder is detailed in the ESI (Fig. S7†), which shows a strong radial dependence. Preferential upward or downward emission can thus further be controlled by placing QDs selectively on the center or edge of the cylinder.

## Conclusions

In conclusion, we demonstrate accurate control over the directional emission of luminescent quantum dots selectively placed on top of resonant Si nanocylinders on a Si substrate. The QDs are placed on the silicon nanostructures using a novel soft-stamp method that selectively prints on the top surface of the nanostructures. The angular distribution of the QD emission is controlled through controlled coupling with Mie-like resonances in the cylinders. By varying the position of quantum dots on the cylinders and by engineering the cylinder diameter, the ratio of upward and downward emission can be strongly controlled. Placing QDs only in the center of the nanocylinder enhances downward emission into the Si substrate, while a homogeneous distribution over the surface creates a strongly enhanced upward emission away from the substrate. The relative ratio of upward or downward emission is dictated by far-field interference of direct emission from the dipoles and resonantly excited cylinder modes and is reflected in consistent changes in LDOS that are probed with the lifetime measurements. The experimental PL intensity and lifetime measurements are in good agreement with FDTD simulation results. Our soft-stamping method for QDs onto nanostructures provides a way to integrate optical emitters with nanophotonic structures, with potential applications in emission control of LEDs, LSCs, and in up- and down-conversion for photovoltaics.

## Methods

### Green's function calculations

The angular dipole emission pattern for a dipole above a Si substrate was calculated using the far-field Green's function formalism according to Appendix D of ‘Principles of Nano-Optics’ by Novotny and Hecht.^31^

### FDTD simulations

The fractions of dipole upward and downwards emission and the angular dipole emission pattern for dipoles above Si cylinders were calculated using finite-difference time-domain (FDTD) calculations performed in Lumerical FDTD Solutions.^35^ Perfectly Matched Layer (PML) boundary conditions were used in three dimensions. A single, monochromatic electric dipole source was used for each simulation, with a polarization axis along either the *X*, *Y*, or *Z*-axis. A “scat_ff” power monitor box was used to collect the electric and magnetic field components surrounding the nanocylinder and dipole. Convergence was found at a uniform mesh size of 5 nm, a distance of 250 nm from the structure to each FDTD box boundary, and conformal mesh refinement. To convert the simulated near fields to far field radiation intensities, we used the open-source RETOP tool.^36^ The real value of the optical constants of Si was used (the imaginary part was set to zero).^37^

To multipole decomposition as in Fig. 1g was performed by calculating the electric field inside the nanocylinder according to the method by Evlyukhin *et al.*^34^

Radiative lifetime simulation results were obtained from the same dipole emission simulations by keeping a fixed electric dipole amplitude in each simulation. In this fashion, the total emitted power at constant dipole amplitude is directly proportional to the Purcell factor and the LDOS.^31^ In turn, the inverse of the total emitted power is a measure for the experimental radiative lifetime. Direct comparison of simulated and measured results was possible because the flat Si reference case normalizes both. The absolute values for the LDOS in Fig. 6d were obtained by multiplying the calculated Purcell factor by the free-space LDOS at the emission wavelength:
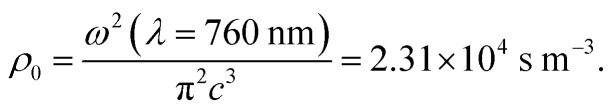


The excitation rate enhancement due to resonant coupling at the pump wavelength was simulated, and the results were used to normalize the experimental photoluminescence intensity and lifetime results. For both laser wavelengths, the electric field intensity above the nanocylinder was determined. We used a monochromatic plane-wave source above a nanocylinder, employing periodic boundary conditions in the substrate plane to mimic the array in the experiment and PMLs in the lateral directions. Using a field monitor, we obtained the field intensity 5 nm above the cylinder surface (see Fig. S3 and S4†). Again, normalization to the flat Si reference case allowed for direct comparison with measurements.

### PDMS chemicals

Elastosil RT 601 A/B (RTV-2 silicone rubber) polydimethylsiloxane (PDMS) was bought from Wacker Chemie. Octadecyltrichlorosilane (ODTS) and 1*H*,1*H*,2*H*,2*H*-perfluorooctyltrichlorosilane were purchased from Sigma Aldrich.

### PDMS stamp preparation

The PDMS stamp was made *via* mixing the prepolymer and curing agent in a ratio of 9 : 1. A plastic rod was used to mix the liquid thoroughly. The viscous liquid was kept in vacuum for 30 min to eliminate the air bubbles trapped due to mixing. A 1 mm thick Viton spacer (made by Speedy 400 laser machine from Trotec Laser B.V.), which defines the final PDMS dimensions, was kept on a fluorosilanized (see below) regular microscopic glass slide. The liquid mixture was poured into the center of the spacer. A UV-ozone activated (45 min in a UV ozone ProCleaner (BioForce Nanosciences)) square glass piece with 15 mm in length and 1 mm in thickness was pressed on the liquid mixture. This assembly was cured in an oven at 80 °C for 24 hours.

### PDMS silanization

For silanization, the sample was activated with oxygen plasma for 10 s. Then the sample was placed beside the desired silane molecule solution in a Teflon boat at a controlled temperature in a Vacucenter VC20 vacuum oven from Salvis LAB, at 50 mbar for a well-defined time. For fluorosilanization of a microscopic glass slide, 10 μL of 1*H*,1*H*,2*H*,2*H*-perfluorooctyl-trichlorosilane was used and placed in the oven at 50 °C for 1 hour. The treatment formed a self-assembled monolayer (SAM) of fluorinated silane chains on the glass slide's surface and rendered it highly hydrophobic. This facilitated peeling off the cured PDMS. For the silanization of cured PDMS, 10 μL of ODTS was used and placed in the oven at 100 °C for 3 hours. This treatment formed SAMs of ODTS on the surface of the PDMS. The layer of ODTS improved the wetting of octane, forming an even layer of QDot 800 *via* spin coating, and prevented swelling of PDMS.

### QD printing

The purchased QDot 800 particles were dispersed in decane. A 100 μL QD solution was mixed with 100 μL of isopropanol and centrifuged at 8000 rpm for 10 min to form a solid precipitate of particles at the bottom of the centrifuging tube. The supernatant was discarded completely, and fresh octane was added to disperse the precipitate: 25 μL for the thick QD print (Fig. 4a) and 100 μL for the thin QD print (Fig. 4b). This solution was spin-coated on the silanized PDMS at 2500 rpm with 650 rpm s^−1^ for 120 s.

The printing experiments were performed with a Universal Testing System model 5965 with 50 kN force capacity from INSTRON. The Si substrate with cylinders was activated for 10 min by UV-ozone treatment. Both the stamp and the substrate were mounted *via* vacuum on the printing machine.

### Atomic force microscopy

AFM images were obtained with a ScanAsyst-AIR probe (Bruker, nominal tip radius 2 nm), operated in PeakForce Tapping mode using a Bruker Dimension Icon AFM.

### Photoluminescence measurements

For the photoluminescence (PL) measurements, a WITec alpha300 RS confocal microscopy setup was used in reflection mode with 20× and 100× magnification, air objectives. The QDs were excited with a 532 nm excitation wavelength continuous-wave laser, 10 mW power, and ∼1 μm-diameter spot size. Spectra were collected using the fiber-connected WITec UHTS spectrometer, where the collection by the fiber acts as the confocal pinhole. Given a fiber core of 100 μm in diameter, we calculate collection spots of 5 (20×) and 1 (100×) μm using FWHM = *d*_fiber_/*M*, with *M* the magnification of the objective.^38^

### TCSPC measurements

The time-resolved PL traces were measured using a home-built time-correlated single-photon counting (TCSPC) setup with a laser at 485 nm excitation wavelength (PicoQuant LDH-D-C-485). The laser repetition rate was 1 MHz. The laser was focused with a Nikon 60× water immersion objective (PlanAPO VC 60× A/1.2 WI) onto the sample. The TCSPC map was created by scanning the sample in a 10 by 10 μm window with a scanning piezo-electric stage (PI-P-733.3CL). The PL was then collected through the same objective, and the laser excitation was filtered with a 488 nm notch filter and a 500 nm long-pass filter. The detectors are silicon-single photon avalanche detectors (Micro Photon Devices, MPD-5CTD) controlled by a PicoQuant HydraHarp 400 event timer.

### Lifetime fitting

We used Wolfram MATHEMATICA 12 (ref. 39) to fit a sum of 2 exponential functions to the data, using the built-in NonlinearModelfit function.

## Author contributions

T. V., B. D., B. E. and A. P. conceived the project. T. V. performed the FDTD simulations, PL measurements, data analysis, and wrote the original draft. B. D. and T. V. performed the TCSPC measurements. B. D. fitted the lifetimes. H. A. and T. V. developed and performed the stamping procedure, under the supervision of E. C. G. H. A., B. D. and T. V. performed the SEM imaging. M. A. performed the AFM measurements, under the supervision of E. A. L. B. E. and A. P. supervised the project. J. v. d. G. and A. P. reviewed and edited the manuscript. All authors provided feedback and contributed to the manuscript.

## Conflicts of interest

There are no conflicts to declare.

## Supplementary Material

NA-004-D1NA00630D-s001
