# Supplementary material for: Directional quantum dot emission by soft-stamping on silicon Mie resonators
Source: Nanoscale Adv. 2022 Jan 7;4(4):1088–97. doi: 10.1039/d1na00630d (PMC8846404; doi:10.1039/d1na00630d)
Supplement: NA-004-D1NA00630D-s001 [file NA-004-D1NA00630D-s001.pdf]

Electronic Supplementary Material (ESI) for Nanoscale Advances.
This journal is © The Royal Society of Chemistry 2022

Supplementary information

As is the case for all plots in the main text, in the following supplementary figures the distance between the dipole and the Si surface is kept constant at 5 nm, and the emission intensity is averaged over all polarizations.

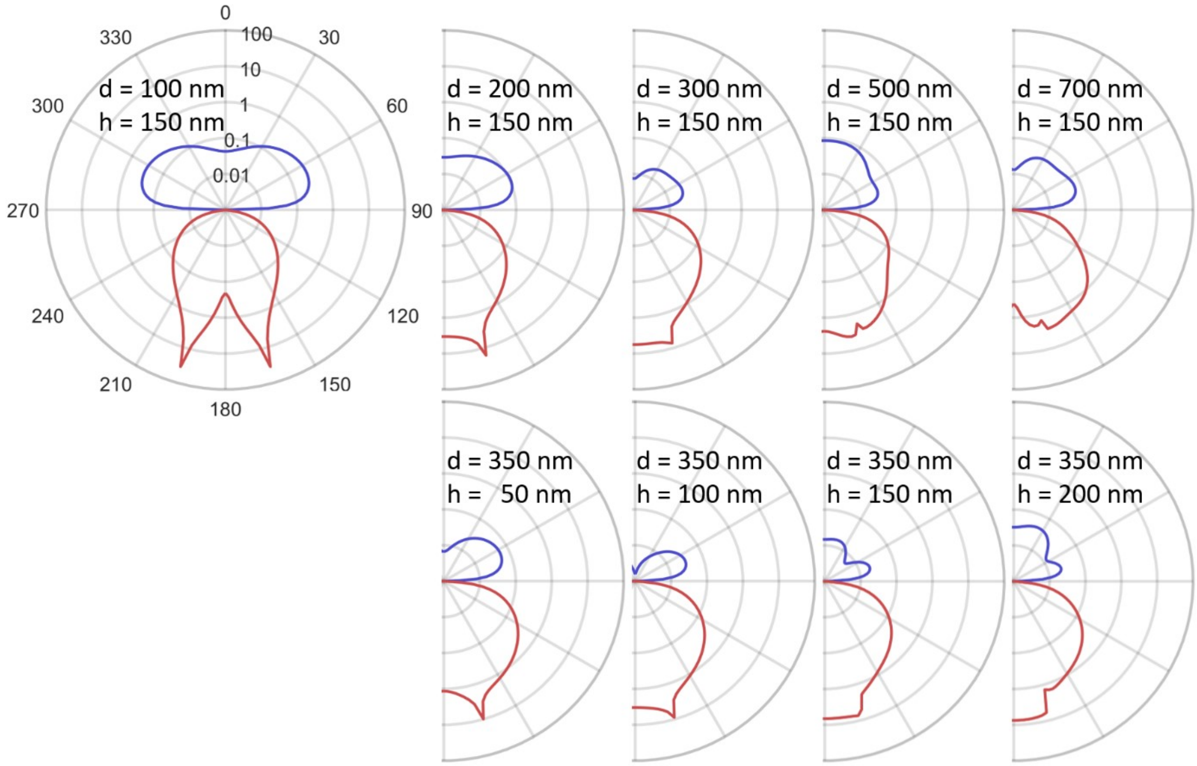

Figure S1: *Calculated azimuthal angular dipole emission patterns for several combinations of cylinder dimensions on a logarithmic radial axis.*

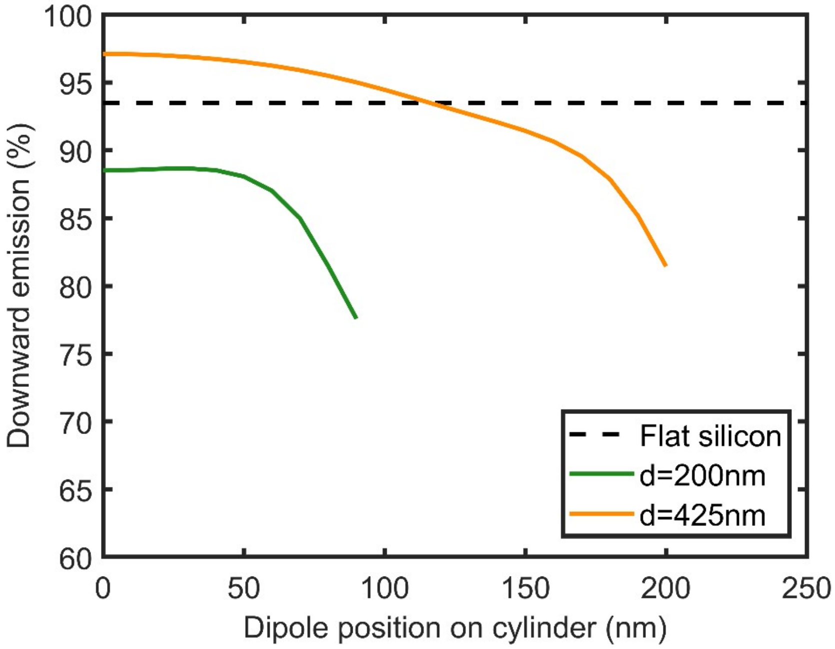

Figure S2: *Similar to Figure 1h: Downward emission percentage as a function of dipole position on the cylinder (0 is at the center of the cylinder).*

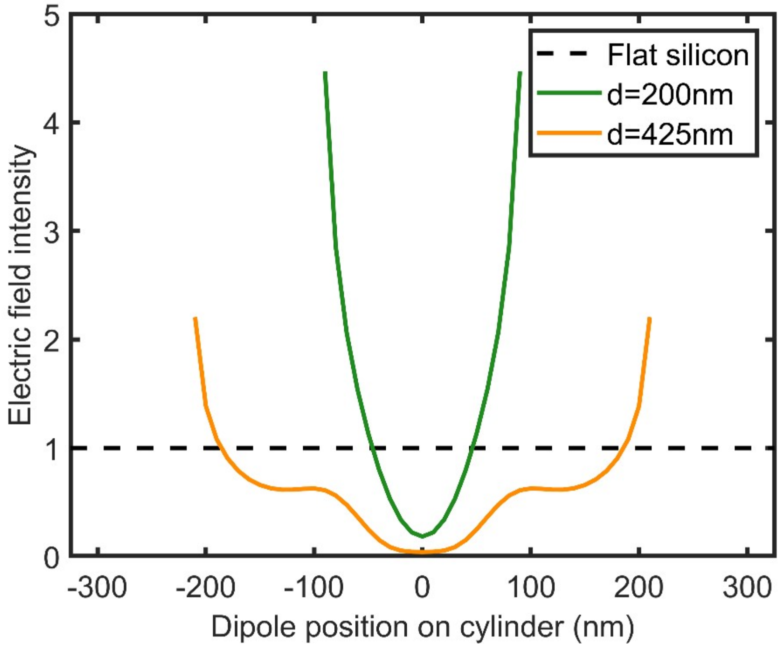

Figure S3: *Excitation enhancement at 532 nm laser wavelength, as a function of dipole position on the cylinder (0 is at the center of the cylinder).*

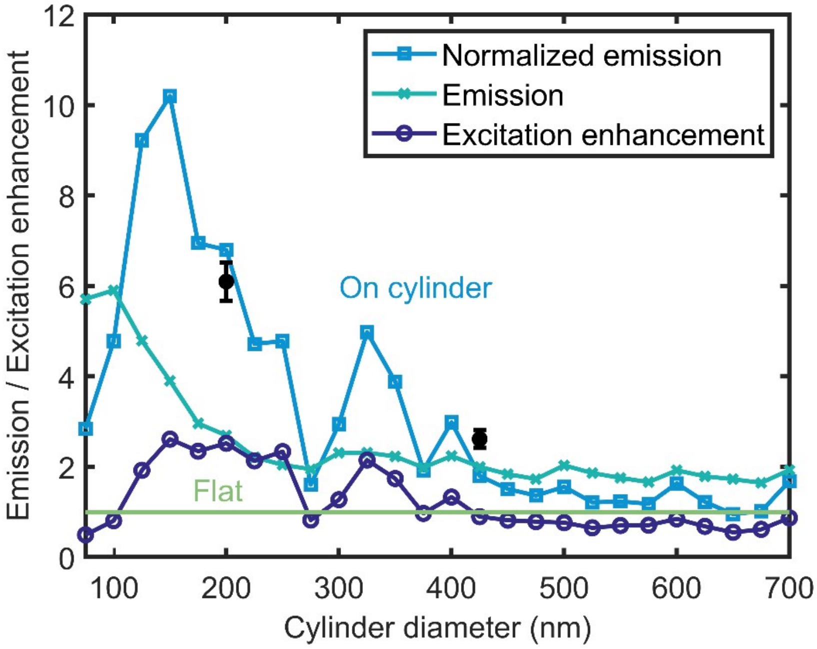

Figure S4: *Similar to Figure 5c: Calculated upward emission intensity (blue) as a function of cylinder diameter at the experimental cylinder height (145 nm), averaged over the cylinder surface. Calculations are corrected for resonant excitation enhancement of the QDs at the cylinder surface at the laser wavelength and normalized to the flat Si reference case (green) to emulate experimental conditions.*

The emission (light blue, cross) and excitation enhancement (dark blue, circle) curves are plotted for reference, showing that the oscillation in the normalized emission curve is mostly due to the excitation enhancement correction.

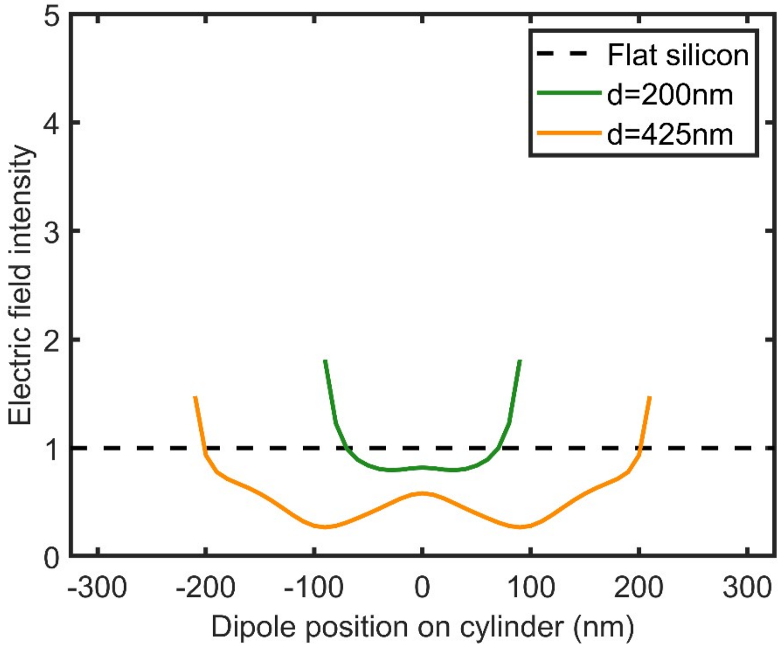

FigureS5: *Excitation enhancement at 485 nm laser wavelength, as a function of dipole position on the cylinder (0 is at the center of the cylinder). Cylinder height is 145 nm.*

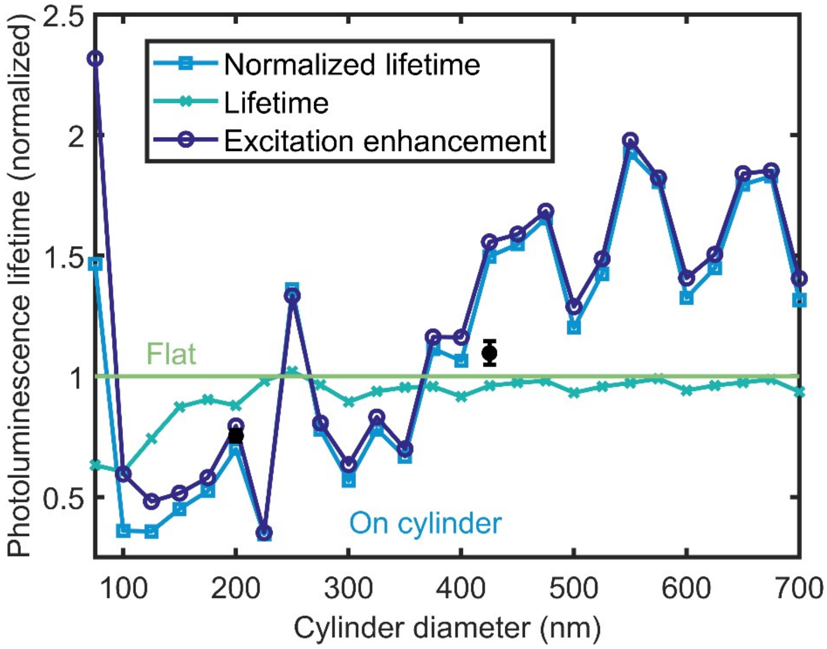

Figure S6: *Similar to Figure 6c: Calculated PL radiative lifetime (blue) as a function of cylinder diameter at the experimental cylinder height (145 nm), averaged over the cylinder surface. Average calculations account for the resonant excitation enhancement of the QDs due to the cylinders at the laser wavelength and are normalized to the flat Si reference (green) to emulate experimental conditions. Experimental data points (fast decay components, black) from the measurements in Figure 6(a,b) are in good agreement with the calculations.*

The emission (light blue, cross) and excitation enhancement (dark blue, circle) curves are plotted for reference, showing that the oscillation in the normalized emission curve is mostly due to the excitation enhancement correction.

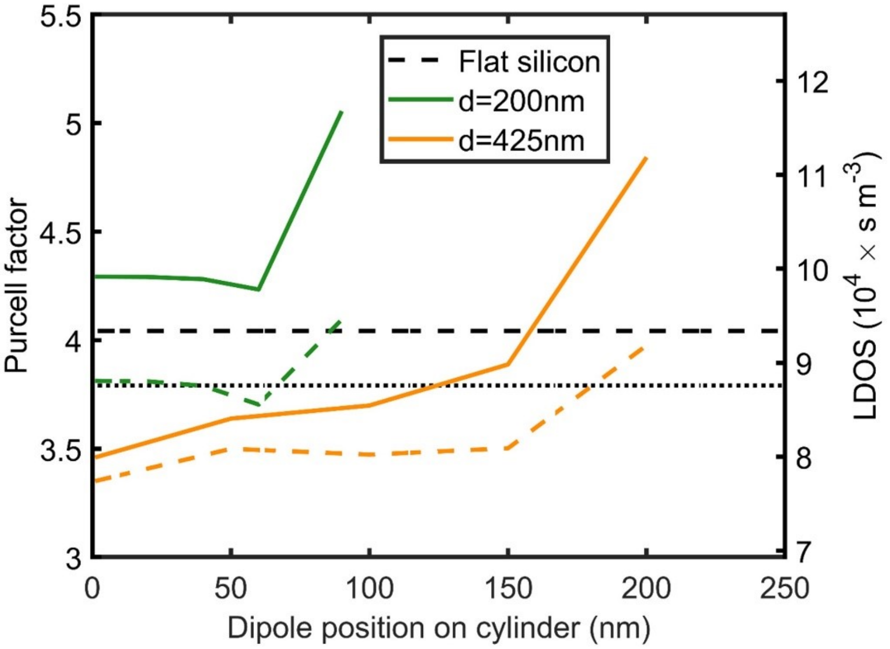

Figure S7: *Similar to Figure 6d: Calculated Purcell factor (left y-axis) and corresponding LDOS values (right y-axis) as a function of dipole position on the cylinder (0 is at the center of the cylinder). Flat Si reference case (black) is indicated, as well as the downward emission fractions (dashed lines).*
